# A Rare Case of Extramedullary Plasmacytoma Presenting as Large Abdominal Mass

**DOI:** 10.7759/cureus.9740

**Published:** 2020-08-14

**Authors:** Dieter Brummund, Benjamin Sinyor

**Affiliations:** 1 Surgery, Aventura Hospital and Medical Center, Aventura, USA

**Keywords:** plasmacytoma, mass, abdomen, pathology, surgery, plasma

## Abstract

A 79-year-old female with a past medical history of multiple myeloma, diabetes mellitus and chronic kidney disease presented to the hospital with generalized weakness. The patient was found to have a firm abdominal wall mass with no underlying skin changes or drainage on physical examination. Computerized tomography of the abdomen and pelvis without contrast revealed a large mass-like structure in the anterior abdominal wall in the subcutaneous region extending into the abdomen through the mesentery with juxtaposition and partial involvement of the left hepatic lobe and distal stomach. Solitary extramedullary plasmacytomas are extremely rare tumors that occur outside of the bone marrow in the absence of systemic involvement. This case reports details the finding of an extramedullary plasmacytomas, which accounts for less than 5% of plasma cell neoplasms.

## Introduction

Out of all the hematologic malignancies in the United States, multiple myeloma accounts for approximately 17% [1]. This equates to approximately 1% of all cancers, with an annual incidence of approximately 4-5 per 100,000 [2]. Multiple myeloma is understood as a disease of older adults, with a slightly higher incidence in African Americans compared to Caucasians, and a lower risk of incidence in those of Asian and Hispanic descent [3]. A slight familial risk has also been documented, demonstrating a higher risk in patients who have a first degree relative with the disease [4].

Extramedullary involvement is an uncommon occurrence in multiple myeloma with studies citing findings of extramedullary plasmacytomas (EMP) in 7% at diagnosis, and 6% incidence later [5,6]. Extramedullary plasmacytomas generally involve the liver, spleen, and hematopoietic tissues reflecting their lymphoid origin. The presence of extramedullary disease both at diagnosis and later has been associated with poor outcomes [7,8]. Diagnosis is confirmed by histopathology demonstrating plasma cell origin and immunohistochemistry with CD138+ staining. Treatment involves chemotherapy such as bortezomib, radiation, novel biologics, and bone marrow transplant.

## Case presentation

We present a case of a 79-year-old female with a chief complaint of abdominal pain ongoing for two months. The pain was described as ongoing, increasing in intensity, non-radiating, generalized, and diffuse with no other associated symptoms. She also complained of generalized weakness during this time. The patient did not report fever, chills, weight loss, night sweats, headache, chest pain, dysuria, melena, diarrhea, or constipation. She reported a medical history of multiple myeloma diagnosed 2 years ago and currently in remission, chronic kidney disease, diabetes, and an episode of complicated sigmoid diverticulitis. She reported a surgical history of subtotal colectomy with ileorectal anastamotic reconscrution, ventral hernia repair and left nephrectomy. 

The physical exam demonstrated an abdominal wall mass that was firm with no overlying skin changes or drainage.

Blood work found a leukocytosis of 15,800 leukocytes per microliter and a hemoglobin of 8.8 g/dL. Liver function was preserved with an alkaline phosphatase of 128 U/L, alanine transferase and aspartate transferase of 15 U/L and 23 U/L, respectively, total protein of 6.7 g/dL, and albumin of 3.4 g/dL was done given the hepatic involvement seen on tomography.

**Figure 1:**
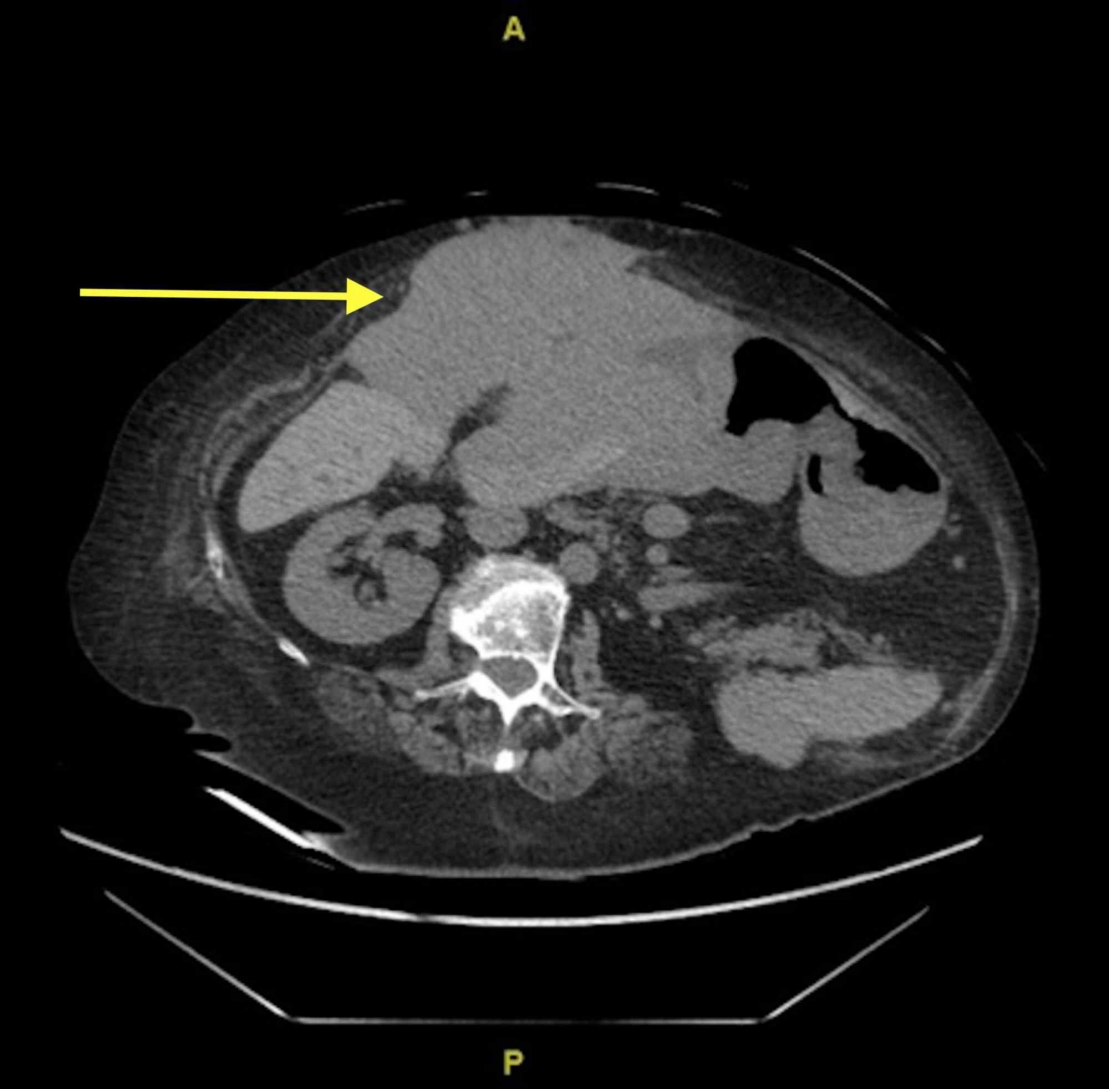
Transverse CT with IV contrast showing a soft tissue mass abutting the anterior liver, infiltrating the anterior abdominal wall and involving the stomach

**Figure 2:**
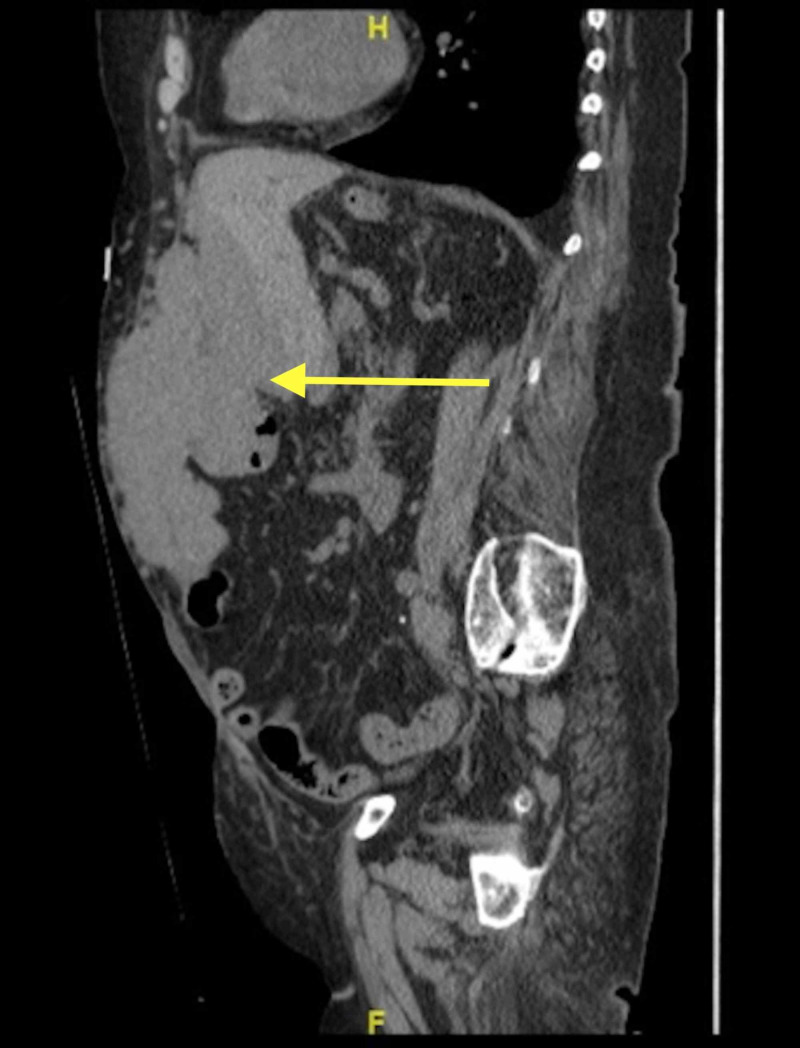
Sagittal CT with IV contrast showing a soft tissue abutting anterior liver, and infiltrating anterior abdominal wall.

Computed Tomography of the abdomen and pelvis without IV contrast reported a large mass-like structure was seen in the anterior abdominal wall in the subcutaneous region extending into the abdomen and through the mesentery with juxtaposition and partial involvement of the left hepatic lobe and distal stomach. The mass measured 17.9 x 15.9 x 8.5 cm and was of soft tissue density with circumscribed margins, 38 hounsfield units, without adjacent inflammatory changes. Additional masses were seen abutting the posterior right hepatic lobe and spleen, and there was a prominent mass in the mid-mesentery (figure 1, 2).

A core needle biopsy performed by interventional radiology confirmed the presence of monoclonal CD138+ plasmacytoid cells consistent with a plasma cell neoplasm.

Medical oncology was consulted and arranged for outpatient follow up with positron emission tomography to evaluate for active areas of relapse and to begin a triple therapy regimen with daratumumab, pomalidomide and dexamethasone.

## Discussion

Multiple myeloma (MM) is a malignant process that involves a neoplastic proliferation of plasma cells characterized by bone marrow involvement, extensive skeletal destruction, renal dysfunction, and elevated total serum protein levels. As a systemic illness, this disease typically presents with systemic symptoms including anemia, bone pain, elevated renal function tests, and weight loss in the majority of patients.

Extramedullary involvement is uncommon, with studies citing findings of extramedullary plasmacytomas (EMP) of 7% at diagnosis, and 6% incidence later in the course of multiple myeloma [6]. EM involvement has been shown to have less favorable outcomes and shorter overall survival [5,6,7,8]. Extramedullary plasmacytomas generally involve the liver, spleen, and hematopoietic tissues reflecting their lymphoid origin. Postulated mechanisms of extramedullary spread involve decreased expression of cell to cell adhesion glycoproteins and downregulation of normal chemokine receptors [8]. 

A subtype of extramedullary disease is cutaneous multiple myeloma and has been associated with particularly aggressive biological behavior and short survival [9]. Common cutaneous manifestations of MM include leukocytoclastic vasculitis, pyoderma gangrenosum, and vesiculobullous disorders.

Diagnosis of extramedullary plasmaycotoma is confirmed through needle biopsy of the affected tissue with histopathology demonstrating plasmacytoid cells and immunohistochemistry staining for CD138+. 

As the presence of extramedullary plasmacytomas in multiple myeloma, both at diagnosis and later during the disease course, has been associated with poor outcomes, extramedullary recurrence warrants an escalation of therapy with proteasome inhibitor therapy found superior to that of thalidomide alone [10]. In certain younger patients, aggressive chemotherapy followed by bone marrow transplant has resulted in improved outcomes not seen with conventional treatment of recurrent extramedullary disease [8].

In our report, we provide evidence of the need to consider a broad differential and to evaluate for recurrence when assessing solid masses in the setting of known hematologic malignancies.

## Conclusions

The presence of an extramedullary mass is particularly concerning for patient prognosis in the setting of known hematologic malignancy. Consideration of extramedullary plasmacytoma in patients who present with subcutaneous masses demonstrated on exam and with a history of multiple myeloma should raise a high index of suspicion for recurrence and treatment failure. The findings warrant prompt subsequent histologic and immunohistory chemistry confirmation with biopsy, genotyping and escalation to 2nd and 3rd line agents in treatment. Further studies are necessary to determine the optimal course of treatment for patients with this particular presentation given the known and previously documented poor prognosis.
